# Clinical Applications of Micro/Nanobubble Technology in Neurological Diseases

**DOI:** 10.3390/biomimetics9100645

**Published:** 2024-10-20

**Authors:** Parth B. Patel, Sun Latt, Karan Ravi, Mehdi Razavi

**Affiliations:** 1 University of Central Florida College of Medicine, Orlando, FL 32827, USA; pa357753@ucf.edu (P.B.P.); ka804136@ucf.edu (K.R.); 2 Biionix (Bionic Materials, Implants & Interfaces) Cluster, Department of Medicine, University of Central Florida College of Medicine, Orlando, FL 32827, USA; sun.latt@ucf.edu; 3 Burnett School of Biomedical Sciences, University of Central Florida College of Medicine, Orlando, FL 32827, USA; 4 Department of Material Sciences and Engineering, University of Central Florida, Orlando, FL 32816, USA; 5 Biomedical Engineering Program, Department of Mechanical and Aerospace Engineering, University of Central Florida, Orlando, FL 32816, USA

**Keywords:** micro/nanobubbles, ultrasound, drug delivery, neurological diseases, clinical studies

## Abstract

Nanomedicine, leveraging the unique properties of nanoparticles, has revolutionized the diagnosis and treatment of neurological diseases. Among various nanotechnological advancements, ultrasound-mediated drug delivery using micro- and nanobubbles offers promising solutions to overcome the blood-brain barrier (BBB), enhancing the precision and efficacy of therapeutic interventions. This review explores the principles, current clinical applications, challenges, and future directions of ultrasound-mediated drug delivery systems in treating stroke, brain tumors, neurodegenerative diseases, and neuroinflammatory disorders. Additionally, ongoing clinical trials and potential advancements in this field are discussed, providing a comprehensive overview of the impact of nanomedicine on neurological diseases.

## 1. Introduction

Nanomedicine is a rapidly evolving field of medicine examining the use of nanotechnology and nanomaterials in the diagnosis, imaging, monitoring, prevention, and treatment of disease. One growing section of the field focuses on the application of ultrasound to enhance the targeting and drug delivery properties of micro/nanobubble technology [1]. The advent of ultrasound-mediated drug delivery using micro/nanobubble technology marks a significant leap forward in precision medicine, providing a novel solution to the longstanding challenge of targeted therapeutic delivery. Ultrasound waves enhance vascular permeability and facilitate the distribution of drugs through cell membranes into the target tissue’s interstitial spaces [2]. Recently, low-frequency ultrasound has been investigated to improve the delivery and retention of therapeutic-loaded micro/nanobubbles, while high-frequency ultrasound has clinical applications by locally breaking bubbles to release therapeutic agents [3]. Nanotechnology offers substantial benefits for drug delivery, including enhanced targeting capabilities, increased bioavailability, reduced systemic drug effects, controlled drug release, and the incorporation of contrast agents for imaging purposes [4]. Within this realm, micro/nanobubbles are specifically optimized with surface membrane ligands to improve targeting, binding, penetration, and imaging. This innovative technology harnesses the unique properties of micro- and nanoscale bubbles to navigate the body’s complex vascular system, facilitating the direct delivery of drugs to disease sites.

In the context of neurological diseases, overcoming the blood-brain barrier (BBB) is a major hurdle for effective drug delivery. Neurological disorders account for 16.5% of global deaths and 276 million disability-adjusted life-years (DALYs), making them a leading cause of disability worldwide, as reported by the Global Burden of Diseases, Injuries, and Risk Factors Study (GBD) in 2016 [5]. This urgent need for effective interventions highlights the potential of nanotechnology to address significant healthcare demands and improve patient outcomes. Current treatments show limited efficacy, often involve side effects, and suffer from inefficient delivery systems. Recent advancements in ultrasound-mediated drug delivery using micro/nanobubble technology offer innovative solutions to this problem, allowing for the targeted and controlled release of therapeutic agents [6]. Although a wide number of publications exist on the clinical applications of microbubbles [7,8,9,10], there is a lack of recent publications on the clinical applications of both microbubbles and nanobubbles. Even fewer publications exist that can act as a concise reference for further clinical applications and research for micro/nanobubble-assisted ultrasound in neurological diseases. This review focuses on the principles and applications of micro/nanobubble-assisted ultrasound in neurological diseases, highlighting recent clinical trials and future directions in this rapidly evolving field.

## 2. Principles

### 2.1. Micro/Nanobubble Technology

The discussion of microscopic and nanoscopic bubbles often makes the distinction between two types: the bulk bubble and the surface bubble [11]. The focus of this work centers on the bulk bubble, a spherical bubble containing a gas-filled core, as opposed to the surface bubble, a surface-adhered spherical cap with a gas-filled core [12]. Micron-sized bulk bubbles, or microbubbles, are typically defined to be 1–10 µm in diameter [10]. Nano-sized bulk bubbles, or nanobubbles, are typically defined to have diameters less than 100 nm [12], though some sources allow this definition to extend to bubbles with diameters of 1–1000 nm [13]. Unseen in larger, macroscopic-sized bubbles, the micron and submicron diameters of these bubbles induce unique physiochemical properties, such as a high specific surface area, extremely slow rising velocity, and longevity [13,14]. Nanoscopic bubble sizes additionally allow for improved cell permeability and payload internalization into cells [15,16,17]. The choice of gas core and shell contributes to the particular size of the nanobubble, along with a variety of other physical bubble properties, including acoustic capability, stability, coalescence, and gas diffusion rate [18]. Numerous gases have been used for the core of micro/nanobubbles, such as hydrogen for use in gasoline or oxygen for wastewater treatment [19,20]. In biomedical applications, the core is typically composed of an inert, hydrophobic gas, which enhances stability by preventing gas dissolution into the surrounding fluid, thereby avoiding toxicity to the human body [21]. Although micro/nanobubbles can exist without a shell, the addition of an exterior shell further stabilizes the bubble by preventing direct contact with the surrounding solution [16,21]. Moreover, these bubbles have seen widespread use as ultrasound contrast agents, a property attributed to their high echogenicity from the resonance of the shell in addition to the gas core [3,22]. The shell can comprise a variety of inorganic or organic substances, including lipids, proteins, polymers, and surfactants [18,23]. The exterior shell provides the opportunity for further functionalization through external surface conjugation or the protection of internally loaded compounds [2]. Accordingly, drug-release characteristics are also influenced by the choice of gas and shell, affecting bioavailability, cell permeability, and target-site specificity [2,24,25,26]. Overall, microbubbles and nanobubbles are promising platforms for the targeted delivery of therapeutic agents while providing diagnostic capabilities [27,28].

### 2.2. Fabrication and Functionalization

The fabrication of micro/nanobubbles involves creating a stable core-shell structure that can withstand the physiological environment while retaining acoustic capabilities. Zhou et al. proposed two pathways that lead to the generation of stable nanobubbles: bubble nucleation and bubble collapse [29]. Formation by bubble nucleation involves the formation of bubble nuclei due to a decrease in gas solubility from an increase in pressure, typically in a gas-oversaturated solution [13]. Formation by bubble collapse involves the formation of microbubbles and nanobubbles by the breakdown and shrinkage of larger bubbles [29]. Abiding by these principles, commonly used techniques for micro/nanobubble fabrication include cavitation, agitation, membrane dispersion, electrolysis, compression-decompression, and microfluidics [29,30,31,32,33]. Each method can simultaneously produce both microbubbles and nanobubbles at different concentrations and size distributions depending on synthesis parameters [34]. Agitation involves using mechanical methods to disturb a sealed vessel containing bubble shell components dissolved in a solvent and a gas that eventually becomes the core. Agitation yields high bubble concentrations, up to 10^11^ per mL, though resulting bubbles are polydisperse in size [30]. Cavitation leads to bubble nucleation as a result of sudden areas of low pressure, commonly generated either hydrodynamically or acoustically [35]. Acoustic cavitation utilizes ultrasound waves to cause pressure oscillation, generating areas of compression, high pressure, and areas of rarefaction, low pressure [36]. Xu et al. directly compared mechanical agitation and sonication methods, finding that sonication generally produced smaller microbubbles, though mechanical agitation produced more stable microbubbles [37]. However, Nirmalkar et al. found longitudinally stable bulk nanobubble suspensions over several months after synthesis through acoustic cavitation [38]. The concentration of nanobubbles decreased over time, depending on the starting concentration, though they remained detectable for several months after synthesis [38]. Similar to acoustic cavitation, hydrodynamic cavitation causes areas of low pressure due to an increase in flow velocity [39,40]. For the membrane dispersion method, a gas is compressed into a liquid before it is allowed to flow through a porous membrane, leading to the formation, growth, and detachment of micro/nanobubbles [41]. Ultaowski et al. were able to produce nanobubbles that were stable for over a month through the membrane dispersion method [42]. Formation by electrolysis involves using an applied current to generate surface bubbles on the electrode before detaching to form bulk bubbles [43,44]. Kikuchi et al. observed electrolytically produced oxygen nanobubbles to increase in size over time before the disappearance of the nanobubbles by 72 h [45]. Anand et al. found greater stability of electrolytically produced nanobubbles in alkaline solutions, compared to neutral and acidic solutions [43]. Compression–decompression involves the forceful dissolution of a gas into a solution by compression followed by a subsequent slow decompression, generating bulk bubbles [46]. Microfluidics, which is particularly relevant to further understanding micro/nanobubble behavior, involves the precise manipulation of fluids and their flow at a micrometric scale using miniature devices [47,48,49,50]. The advantages and disadvantages of each of these techniques are summarized in Table 1. Paknahad et al. have summarized the resulting bubble size and concentrations from each of these methods, specifically for bulk nanobubble generation [30].

**Figure 1:**
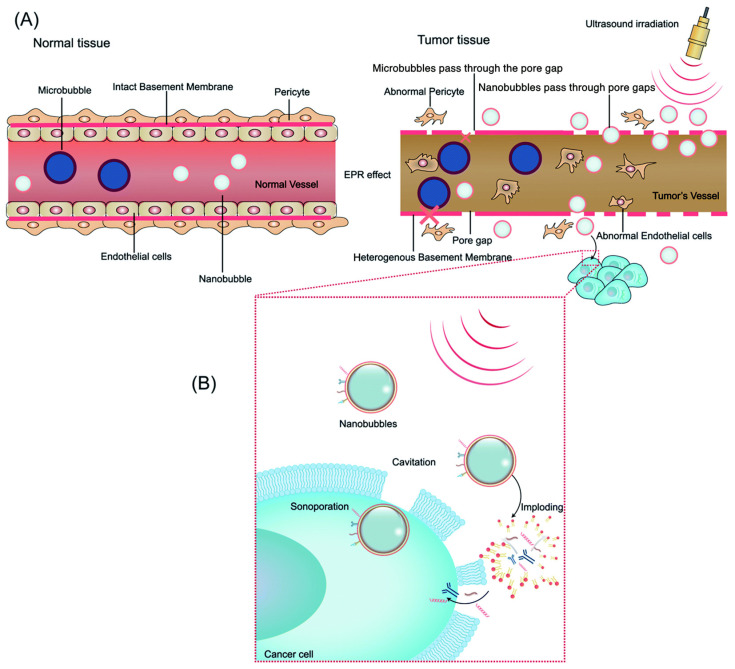
Schematic demonstrating the use of biological factors in ultrasound-mediated bubble delivery and depiction of delivery mechanisms for drugs/genes to targeted tumor tissue. (**A**) Graphic of the Enhanced Permeability and Retention (EPR) effect in tumor and normal tissues. Nanobubbles can pass the endothelial pore in tumor tissue while microbubbles cannot. (**B**) Ultrasound-induced cavitation, sonoporation, increased membrane permeability, and drug release of nanobubbles. Reprinted with permission from Su et al. [53].

However, despite the extensive number of bulk micro/nanobubble synthesis methods, a clear mechanism that explains their stability remains unfounded. According to the Young–Laplace equation, bulk micro/nanobubbles are predicted to have a high-pressure differential, conferring thermodynamic instability [12,51]. Some theories postulate that stability arises from either the adsorption of surface contaminants or the pinning of surface charges [29]. Regardless, the existence of long-lived micro/nanobubbles provides the opportunity to use them as nanoplatforms for drug delivery (Figure 1). Additionally, more stable micro/nanobubbles allow for improved long-term drug release and more sustained contrast during extended ultrasound imaging [21]. Methods to improve nanobubble stability, apart from the choice of the initial synthesis method, involve the choice of shell composition, choice of gas core, addition of surfactant, and inclusion of electrolytes [21,52]. A gas core that has a low partition coefficient with the surrounding solution should be chosen to improve the stability of the bubble [38]. The choice of shell composition can control leakage of the gas core and bubble coalescence [53]. Nirmalkar et al. found that the addition of sodium dodecyl sulfate, a surfactant that can reduce bubble surface tension, improved nanobubble recovery after freeze-thawing [38].

In addition, ligands, antibodies, or peptides can be conjugated to the bubble surface to recognize and bind specific cellular receptors [54]. For instance, fucoidan, which has a strong affinity for P-selectin released by platelets during thrombus formation, can be attached to the bubble surface to enhance the specificity and efficacy of thrombolytic agents [54]. As explored in the next section, drug compounds can be encapsulated in the interior of micro/nanobubbles to avoid non-specific interactions while allowing for localized therapeutic effects through eventual release by ultrasound [55].

**Table 1:** Summary of the advantages and disadvantages of nanobubble manufacturing methods.

Preparation Method	Advantages	Disadvantages	References
Mechanical Agitation	- Avoids generating localized areas of high energy and temperature - Capable of producing large scales of bulk bubbles	- Difficult to control the monodispersity of bubbles - Often requires further fractionation to isolate bubble sizes	[23,30,56,57]
Acoustic Cavitation	- Reactive radicals can be generated that can be used for further chemical reactions, such as the creation of polymers. - Modification of ultrasound power and frequency can allow for controllable and predictable bubble sizes.	- Ultrasound can detach nearby solids, leading to contamination. - Yield is limited by the size of the horn, though modifications can be made to improve scalability. - Localized areas of high temperature are generated.	[23,38,58,59]
Hydrodynamic Cavitation	- Allows for real-time control of size through modifying agitation speed - Efficient in both energy and cost - Reactive radicals can be generated through this method.	- High-energy areas are generated, though they are comparably lower compared to acoustic cavitation. - Although this method has excellent scalability, there is a lack of models for predicting cavitation effects that limits commercialization.	[30,34,60]
Membrane Dispersion	- Controllable, monodisperse sizes can be obtained by modifying pore sizes. - Microbubbles and nanobubbles can be synthesized continuously.	- Membranes with small pore sizes, such as Shirasu porous glass membranes, are expensive and fragile. - Although continuous synthesis is possible, many membranes require a low velocity of flow to prevent membrane damage.	[61,62,63]
Electrolysis	- Size and quantity of generated bubbles can be determined by changing current, temperature, operating time, or electrolyte concentration.	- Gas core selection seems to be limited to only electrolytic gases, excluding commonly used inert gases. - There is limited research on the electrolytic generation of drug-loaded bubbles or shelled bubbles.	[30,43]
Compression–decompression	- Relatively simple and accessible method of producing a large number of bulk nanobubbles	- Prone to contaminants from lubricant or material chipping from the container surface - Although decompression time and choice of solution allows for some control over size and monodispersity, other methods provide finer control.	[18,30,64]
Microfluidics	- Capable of producing monodisperse nanoscale particle sizes - Precise regulation of fluid flow can allow real-time size control.	- Microfluidic device fabrication is expensive and time-consuming. - Obstructions in microfluidic channels are difficult to clean.	[30,48,65,66,67]

### 2.3. Principles of Ultrasound-Mediated Drug Delivery

**Figure 2:**
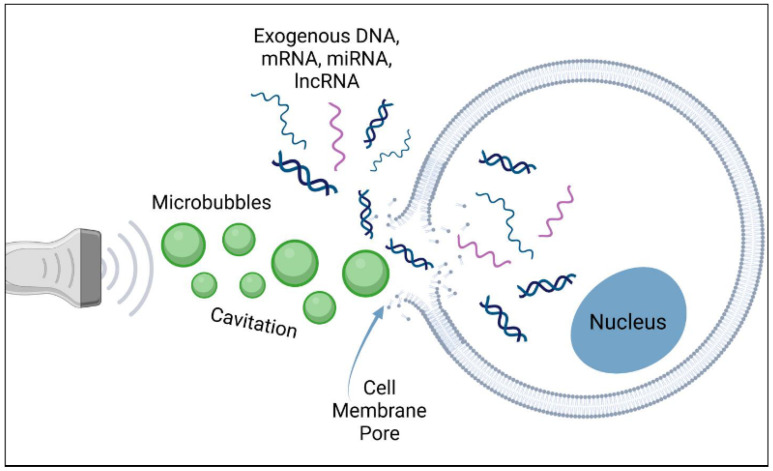
Schematic depicting ultrasound-induced formation of a cell membrane pore via cavitation of microbubbles, allowing diffusion of exogenous contents such as nucleic acids. Reprinted with permission from Krut et al. [71].

Ultrasound remains a staple in medical diagnostic imaging, standing as the second most-used clinical imaging technique behind the X-ray [68]. Though ultrasound as a diagnostic tool has been thoroughly explored, the applications for the use of ultrasound as a therapeutic mechanism are rapidly expanding, especially with the advent of new micro/nanotechnologies [69,70]. Given the addition of therapeutic applications to diagnostic ultrasound, it can now be considered a “theranostic” tool, integrating both diagnostic and therapeutic properties (Figure 2).

#### 2.3.1. Nanobubble Applications in Imaging

With ultrasound technology, micro/nanobubbles are also capable of acting as contrast agents to provide enhanced imaging [72]. Contrast agents are particularly useful during the assessment of microvasculature, which is difficult to visualize with traditional ultrasound techniques alone [73]. Microbubbles are widely used in clinics as an ultrasound contrast agent, producing a stronger signal due to a mismatch in acoustic impedance between the bubble and the host tissue [74]. Common compositions of microbubbles for use as ultrasound contrast agents involve a gas core with low solubility, such as nitrogen or perfluorocarbon, with a biodegradable shell [75]. However, recent studies have demonstrated that nanobubbles can also have promising acoustic potential after improvements in synthesis methods have led to narrower size distribution and greater control over shell interfacial tension [76].

#### 2.3.2. Nanobubble Applications in Drug Delivery

Furthermore, ultrasound can increase the bioactivity of micro/nanobubbles, primarily through two methods: the permeabilization of membrane barriers and the targeted destruction of micro/nanobubble drug carriers [77,78,79]. Sonoporation (Figure 1 and Figure 2) refers to the generation of pores in the cell membrane due to two types of cavitation caused by the interaction of ultrasound with micro/nanobubbles [79,80]. Stable cavitation leads to small-amplitude oscillations in the bubble that disrupt the cell membrane in a push–pull manner, and inertial cavitation induces implosion of the bubble, leading to the formation of microjets to disrupt the cell membrane [81]. Inertial cavitation occurs at higher levels of ultrasound pressure than those of stable cavitation, typically inducing larger sites of sonoporation [81,82]. Moreover, inertial cavitation is the mechanism responsible for the collapse of the micro/nanobubble and the subsequent release of a loaded drug [2,83]. Therefore, the combined sonoporation and targeted therapeutic release effects of ultrasound-triggered micro/nanobubble release provide the potential for the enhanced localized penetration of biological barriers (Figure 2). Several studies have already demonstrated improved drug delivery efficiency across the barrier using this technology [84,85].

## 3. Current Clinical Applications

### 3.1. Stroke

#### 3.1.1. Ischemic Stroke

Ischemic stroke, caused by a blockage in blood vessels supplying the brain, leads to neuronal death and is a leading cause of morbidity and mortality worldwide. Current treatment guidelines recommend IV thrombolytic therapy, such as recombinant tissue plasminogen activator (tPA) or tenecteplase (TNK), for ischemic stroke presenting within 4.5 h of symptom onset or endovascular thrombectomy to those not eligible or presenting beyond 4.5 h of symptom onset. These are the only FDA-approved treatments for reestablishing cerebral blood flow in ischemic stroke patients, but many challenges exist, including a narrow therapeutic time window for thrombolytics, potential risk of cerebral hemorrhage, ischemic-reperfusion injury, neurotoxic effects of tPA, and risks associated with endovascular thrombectomy, if that is performed [54]. Micro/nanobubbles loaded with thrombolytic agents can be directed to the clot site using ultrasound, enhancing the delivery and efficacy of the treatment while minimizing systemic side effects. Recent in vivo and in vitro research has demonstrated that this approach has the potential to increase the treatment window for ischemic stroke and improve patient outcomes by facilitating more effective clot dissolution. However, more clinical trials are needed to clarify the utility of sonothrombolysis for ischemic stroke management [86]. It is also possible to include additional therapeutics within these functionalized bubbles to aid in ischemic stroke management, such as anti-inflammatory, neuroprotective, antioxidant, or imaging agents.

#### 3.1.2. Clinical Studies

Although there has been preclinical and clinical research for other medical applications of nanoparticle technology, their potential in the treatment of stroke has yet to be investigated. Regarding atherosclerosis generally, clinical and preclinical studies have focused on acute conditions like deep vein thrombosis and pulmonary embolism, showing that ultrasound-mediated delivery can significantly reduce clot size and improve blood flow with lower doses of thrombolytics, reducing the risk of bleeding complications [87]. Experimental approaches are investigating the delivery of anti-inflammatory agents, cholesterol-lowering drugs, or genes encoding for therapeutic proteins directly to atherosclerotic plaques, aiming to prevent plaque progression or induce regression. Following myocardial infarction (MI), targeted micro/nanobubble-mediated delivery can be used to transport stem cells, growth factors, or other regenerative agents directly to the damaged heart tissue. Research has also explored the use of this technology for enhancing the efficacy of stem cell therapy in post-MI cardiac repair, with studies showing improved stem cell engraftment and reduced infarct size. Preclinical studies have also demonstrated that ultrasound-mediated delivery of angiogenic factors (VEGF, FGF) using micro/nanobubbles can enhance neovascularization in ischemic limbs or cardiac tissue, improving tissue perfusion and function [88,89].

### 3.2. Oncology

#### 3.2.1. Brain Tumors

**Figure 3:**
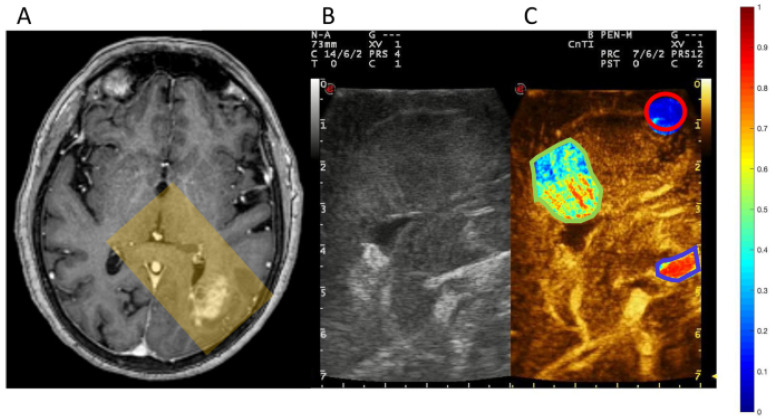
Qualitative assessment of microbubble concentration in specific brain areas for a case involving parietal glioblastoma: (**A**) Gadolinium-enhanced T1-weighted axial MR demonstrates the tumor. The translucent yellow rectangle highlights the insonation plane through the craniotomy, aligning with the ultrasound (US) images. US monitors screenshots of low mechanical index B-mode imaging (**B**) and contrast-enhanced ultrasound (CEUS) images (**C**). In (**C**), three regions are outlined in blue (artery), red (white matter), and green (tumor), demonstrating microbubble density using a JET color scale. Reprinted with permission from Prada et al. [91].

One of the primary applications of micro/nanobubble technology in oncology is to enhance the delivery of chemotherapeutic agents directly to tumor sites. The encapsulation of chemotherapy drugs within micro/nanobubbles allows for targeted delivery, minimizing systemic exposure and, consequently, reducing side effects. Upon reaching the tumor site, focused ultrasound induces bubble cavitation, resulting in the localized release of the chemotherapeutic agent. This process not only increases the drug concentration within the tumor but also improves penetration into the tumor microenvironment, potentially overcoming the issues of poor drug diffusion and heterogeneous distribution seen with conventional chemotherapy [90]. Glioblastoma and other malignant brain tumors pose significant treatment challenges due to the BBB and the tumors’ resistant nature. Ultrasound may increase the permeability of the BBB and aid in the concentration, targeting, and release of micro/nanobubble-encapsulated therapeutics (Figure 3) [2].

#### 3.2.2. Clinical Trials

**Figure 4:**
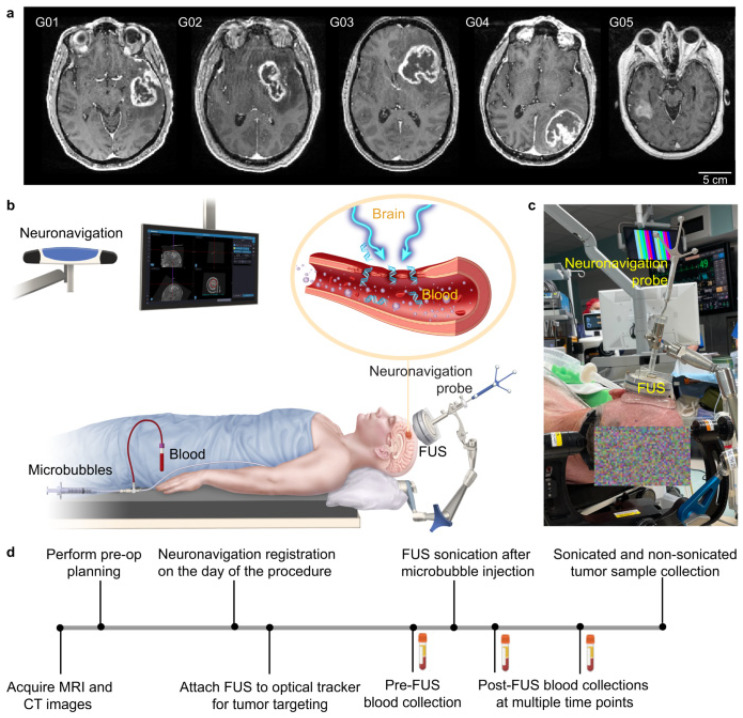
(**a**) T1-weighted MRI images of the five patients enrolled in the study before sonobiopsy. (**b**) Picture of the sonobiopsy and neuronavigation system. (**c**) Image of the FUS transducer paired with a neuronavigation probe used during sonobiopsy. (**d**) Sonobiopsy procedural steps. Reprinted with permission from Yuan et al. [92].

**Figure 5:**
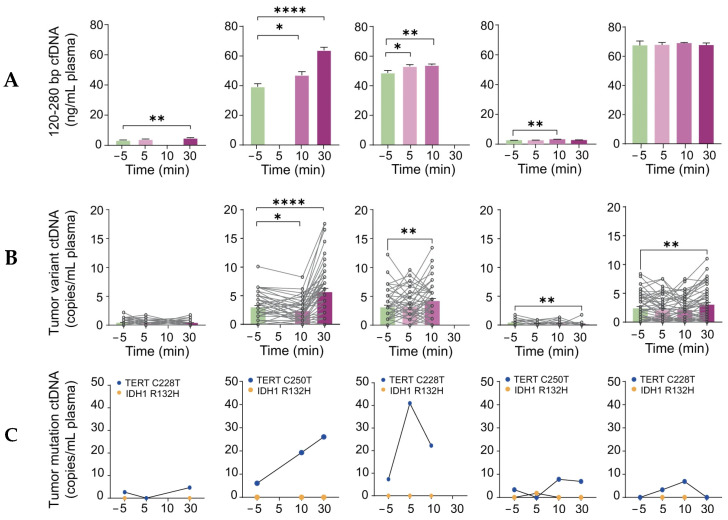
Calculation of the absolute level of patient-specific tumor variant ctDNA in the plasma samples (tumor variant ctDNA copies/mL plasma) pre- and post-FUS. (**A**) Blood concentrations of plasma single nucleosome length cfDNA (fragments of 120-280 bp) collected after pre-FUS and post-FUS. Data in bar graphs are represented as means ± standard error of the mean. An ordinary one-way ANOVA with an uncorrected Fishers’ LSD comparing post-FUS time points versus pre-FUS time points was performed. * *p* < 0.05, ** *p* < 0.01, and **** *p* < 0.0001. (**B**) A personalized tumor-informed ctDNA assay was used to assess plasma patient-specific tumor variant ctDNA concentrations. Data in bar graphs are represented as means ± standard error of the mean. A repeated measures one-way ANOVA with an uncorrected Fishers’ LSD comparing post-FUS time points versus pre-FUS time points was performed. * *p* < 0.05, ** *p* < 0.01, and **** *p* < 0.0001. (**C**) ddPCR was used to detect the mutation level of TERT and IDH1. The data shown represent the time points for when the blood samples were drawn. The results showed that sonobiopsy increased the detection of patient-specific tumor variant ctDNA in G02’s, G03’s, and G05’s plasma. G03 reached the highest level of plasma tumor variant ctDNA at 10 min post-FUS compared with pre-FUS, while G05 reached the highest level of plasma tumor variant ctDNA at 30 min post-FUS compared with pre-FUS. Reprinted with permission from Yuan et al. [92].

A landmark study conducted by Yuan et al. was performed to assess the feasibility and safety of sonobiopsy in increasing circulating plasma levels of tumor biomarkers in high-grade glioma patients [92]. The BBB limits the release of brain tumor-specific biomarkers into the peripheral circulation. Biomarkers such as DNA, RNA, protein, and extracellular vesicles provide a minimally invasive, fast, and cheap alternative for diagnosing and monitoring tumors. By applying transcranial low-intensity focused ultrasound (FUS) to the BBB, the oscillation and subsequent cavitation of the bubbles temporarily disrupt the tight endothelial cell junctions that form the barrier. This disruption creates temporary openings in the BBB, allowing for the selective and enhanced permeation of biomarkers. This study used an affordable and compact FUS device (Imasonics, Voray-sur-l’Ognon, France) for targeted sonobiopsy and localized tumor biomarker release (Figure 4). Sonobiopsy successfully increased the circulating levels of ctDNA without causing observable tissue damage after the histological analysis of a resected tumor (Figure 5).

This study found no pathological evidence of tissue damage or even short-term inflammatory/immune response from sonobiopsy. Transcriptome analysis revealed that differentially expressed genes were only 0.19% of the total identified genes after FUS. Most upregulated and downregulated genes were linked to the cell’s physical structures, such as cell interactions and the extracellular matrix, so it is unlikely that sonobiopsy had any lasting deleterious genomic effects. This prospective study indicates that sonobiopsy can be combined with blood-based biomarker analysis for a noninvasive diagnosis of brain tumors without tissue damage. The authors emphasize the unknown safety and long-term effects of repeated disruptions of the BBB. Other considerations include the acute safety of transient BBB opening, precise control over ultrasound parameters and bubble concentration, avoiding therapeutic delivery to healthy brain tissue, as well as long-term safety of bubble accumulation within brain [92].

A phase I clinical trial conducted by Park et al. involved six glioblastoma patients who received multiple cycles of temozolomide (TMZ) chemotherapy combined with MR-guided Focused Ultrasound (MRgFUS) to disrupt the BBB [93]. There were a total of six patients with five receiving six cycles of BBB disruption from MR-guided FUS and one patient receiving three cycles. The mean follow-up time was 15.17 months, and the survival rate at 1 year was 100%. The study found no acute or long-term complications from disruptions of the BBB. Four patients experienced no recurrence of GBM. Two patients experienced recurrence, with one undergoing surgery and the other restarting TMZ chemo with a strong possibility of future surgery. TMZ can partially cross the BBB with concentrations around 20% of plasma levels, and past research shows that FUS may increase brain concentrations by 7.7-fold. More extensive research is required to confirm survival benefit in those genetically susceptible to TMZ treatment. The study showed that all patients tolerated the procedure well, with no significant adverse events. MRI scans confirmed the temporary opening of the BBB, and follow-up studies indicated improved drug delivery and tumor control compared to those of historical controls [93].

### 3.3. Degenerative Diseases

#### 3.3.1. Alzheimer’s Disease

In Alzheimer’s disease, the current accepted theory is that the accumulation of amyloid-beta plaques and tau tangles disrupts neuronal function and leads to severe cognitive dementia. Micro/nanobubbles can facilitate the delivery of antibodies, neurotrophic factors, or gene therapies that target these pathological proteins. Early-phase clinical trials have shown that MR-guided FUS can safely open the BBB, allowing for the delivery of therapeutic agents that may modify disease progression [6,94,95].

#### 3.3.2. Clinical Studies

Lipsman et al. conducted a pilot study where five patients with mild to moderate Alzheimer’s disease underwent MR-guided FUS to transiently open the BBB [6]. The BBB was opened twice in the dorsolateral prefrontal cortex one month apart. The opening and successful closure 24 h later was measured by T1 MRI gadolinium enhancement in the right frontal lobe. There were no serious clinical or radiological adverse effects. Cognitive assessments indicated no changes three months later, and [18F]-florbetaben PET did not find changes in amyloid deposition post-sonification [6]. This safety and feasibility study provided data for subsequent larger trials to explore whether BBB opening via focused ultrasound alone, or combined with a therapeutic, can reduce amyloid deposition or provide clinical benefit to Alzheimer’s patients. Rezai et al. performed another clinical trial involving BBB opening via FUS of the hippocampus and entorhinal cortex in six patients with early Alzheimer’s disease [95]. Post-FUS contrast MRI enhancement of the hippocampal parenchyma confirmed the opening and subsequent closing of the BBB 24 h later. There were 17 total sessions across all patients that were well-tolerated with no clinical, cognitive, or radiological adverse effects on follow-up. Notably, a follow-up study was conducted and found amyloid plaque reduction from FUS-mediated BBB opening alone. A reduction in plaque was found in the treated hippocampus compared to the untreated contralateral hippocampus as showed by the [18F]-florbetaben PET analysis. While BBB opening through FUS has been shown to reduce amyloid plaque burden in animal studies, this is the first to show similar findings in a human trial [94].

#### 3.3.3. Parkinson’s Disease

Parkinson’s disease, characterized by the loss of dopaminergic neurons, has been another focus of ultrasound-mediated drug delivery. Studies have explored the delivery of neuroprotective agents and gene therapies using micro/nanobubbles, showing promise in preclinical models [96]. Clinical trials are underway to assess the safety and efficacy of these approaches in patients.

#### 3.3.4. Clinical Studies

In a phase I clinical trial, seven patients received MR-guided FUS to assess the safety and feasibility of opening the BBB at the parieto–occipital–temporal junction in patients with Parkinson’s disease. The procedure was well-tolerated by all patients with no clinical or radiological adverse effects. The closing of the BBB was confirmed within 24 h by the disappearance of gadolinium enhancement. There were no changes in FDG or [18F]-florbetaben PET radiotracer uptake pre- and post-sonification. Surprisingly, cognitive assessments showed mild improvement in the MoCA test, short- and long-term memory, and executive and visuospatial functioning 3–4 weeks after the second treatment. There was no identifiable pattern to the cognitive improvements amongst these patients. No changes were found in the motor and non-motor clinical manifestations of Parkinson’s disease. The study highlights the safety, efficacy, and feasibility of transiently opening the BBB in brain regions affected by Parkinson’s and the future possibility of ultrasound-assisted drug delivery (neurotrophins, antibodies, anti-inflammatories, etc.) to brain regions to slow the progression of Parkinson’s disease [96].

### 3.4. Other Neurological Diseases

Autoimmune disorders affect millions of individuals in the United States. Current treatments are typically non-curative and consist of broad immunosuppressants and low-dose chemotherapeutics. These approaches can weaken the immune system, increasing a patient’s risk of developing infections [97]. This presents a significant opportunity for the application of nanomedicine.

#### 3.4.1. Myasthenia Gravis

Myasthenia gravis (MG) is a rare autoimmune disease where the immune system produces antibodies that attack receptors at the neuromuscular junction, impairing muscle contraction and leading to muscle weakness and fatigue. Current treatments, such as cholinesterase inhibitors and glucocorticoids, focus on managing symptoms, while immunomodulators must be used cautiously due to their significant side effects. Extracellular vesicles (EVs) have emerged as an advanced drug delivery system with the potential to uniquely target tissues compared to traditional nanocarriers like liposomes [97].

#### 3.4.2. Clinical Studies

A preclinical study by Zhou et al. found that EVs loaded with caspase-1 inhibitors (EVs-VX-765) primarily target macrophages, effectively slowing experimental autoimmune myasthenia gravis and showing superior therapeutic effects and reduced toxicity compared to standard VX-765 treatments [98]. Other nanocarriers, such as liposomes, ethosomes, niosomes, lipid-based nanoparticles, and polymeric particles, have been effectively used to deliver immunosuppressive drugs for psoriasis, enhancing their therapeutic effectiveness in both laboratory and animal studies [99]. While immunotherapy remains a cornerstone in the treatment of myasthenia gravis, utilizing nanocarriers such as micro/nanobubbles with greater therapeutic effects and reduced doses, as demonstrated in other diseases, may serve as effective tools for treating MG.

#### 3.4.3. Amyotrophic Lateral Sclerosis (ALS)

ALS is a neurodegenerative disease affecting motor neurons. Focused ultrasound with micro/nanobubbles has the potential to deliver therapeutic agents that may protect motor neurons or modulate the immune response. Early clinical trials have shown the feasibility of this approach, but further research with larger trials and administered therapeutics is needed [100].

#### 3.4.4. Clinical Studies

Abrahao et al. conducted a phase I trial using MR-guided FUS to open the BBB in ALS patients, specifically at the primary motor cortex [100]. The study found a successful BBB opening at the target site via gadolinium leakage at the primary motor cortex. The procedure was safe and well-tolerated, with no significant side effects reported. The transient opening of the BBB at the target site normalized 24 hrs later with no clinical, radiological, or EEG adverse events. The authors emphasize the need for larger trials combining MRgFUS with therapeutic agents to evaluate efficacy and potential benefits in slowing disease progression [100].

## 4. Clinical Trials in Progress

Several clinical trials are currently investigating the efficacy and safety of ultrasound-mediated drug delivery in neurological diseases. For example, ongoing studies are exploring the use of focused ultrasound to enhance the delivery of chemotherapeutic agents in glioblastoma, as well as neuroprotective and gene therapies in Alzheimer’s and Parkinson’s diseases. One clinical trial (NCT03671889) is investigating the feasibility of focused ultrasound-induced BBB opening in Alzheimer’s patients to facilitate the delivery of therapeutic agents. The ongoing study involved the BBB opening in two-week intervals of eight Alzheimer’s disease patients using a 220 kHz FUS transducer in combination with microbubble contrast agents [101]. Post-treatment MRI revealed extravasation of the microbubble contrast agent into intracerebral veins, and there was a week-long permeabilization of the intraparenchymal veins [101]. No intracranial hemorrhage or serious side effects were observed in any of the eight patients after treatment. In phase I of a phase I/II trial (NCT04528680), seventeen patients with recurrent glioblastoma were treated with albumin-bound paclitaxel and microbubbles in conjunction with low-intensity pulsed ultrasound (LIPU-MB) [102]. The treatment consisted of six cycles of escalating dosages of albumin-bound paclitaxel, administered at six-week intervals [102]. The results from the study indicated enhanced drug penetration across the BBB [102]. The ongoing phase II portion is investigating the use of focused ultrasound to deliver both albumin-bound paclitaxel and carboplatin in conjunction with LIPU-MB in patients with recurrent glioblastoma [102]. Another phase II trial (NCT04118764) is evaluating the safety and efficacy of focused ultrasound for BBB disruption in Alzheimer’s patients, coupled with the delivery of anti-amyloid antibodies. From the same investigators, BBB disruption in transgenic mice expressing human amyloid protein showed reduced amyloid protein after treatment [103]. These results seemed to be reflected in the preliminary results from the phase II trial, which showed a reduction in amyloid PET signal in one patient after treatment [103].

## 5. Challenges and Limitations

Despite its potential, ultrasound-mediated drug delivery via micro/nanobubble technology faces several challenges. The stability and circulation lifetime of micro/nanobubbles in the bloodstream, their potential immunogenicity, and precise control over bubble collapse are critical issues that need addressing. Micro/nanobubbles need to remain stable in the bloodstream long enough to reach their target site. However, bubbles can be rapidly cleared by the mononuclear phagocyte system (MPS), reducing their efficacy. Strategies to enhance bubble stability include surface modifications with polyethylene glycol (PEG) to evade immune detection and the use of more stable gas cores, like perfluorocarbons [2]. The introduction of foreign materials can trigger immune responses, leading to rapid clearance and potential adverse reactions. Surface modifications and the use of biocompatible materials can mitigate these risks, but further research is needed to optimize these strategies. Surface modifications with biocompatible materials such as PEG and phospholipids, as well as the use of endogenous materials like albumin for bubble construction, can minimize immune recognition and enhance biocompatibility [2]. Precise control over bubble collapse and drug release is crucial to avoid off-target effects and ensure that the therapeutic agents reach the intended site. Advances in ultrasound technology, such as the development of high-precision imaging and targeting systems, are needed to improve the accuracy and safety of these treatments. These technologies enable clinicians to monitor bubble behavior and drug release in real-time, reducing the risk of off-target effects and enhancing treatment efficacy [89]. Another important challenge is tissue acoustic attenuation, which occurs when tissue absorbs and reduces the ultrasound intensity reaching the nanobubbles. It is essential to measure this attenuation in preclinical studies and optimize the ultrasound dosage to ensure it remains within a safe and effective range. Research is ongoing to develop more stable, less immunogenic, and precisely controlled formulations of micro/nanobubbles. For instance, double emulsions and the incorporation of stabilizing agents in the shell are being explored to extend the circulation time and enhance bubble stability. Additionally, encapsulating bubbles within biodegradable polymers can protect them from premature clearance and improve their delivery efficiency [2].

## 6. Technological and Clinical Advancements

Future research will focus on improving the precision and safety of ultrasound-mediated treatments. This includes refining targeting techniques, optimizing ultrasound parameters, and developing more stable and effective micro/nanobubbles. Additionally, advancements in imaging technologies will enhance the ability to monitor treatment in real time, providing valuable feedback for clinicians [96]. Recent advances in imaging, such as super-resolution ultrasound imaging, allow for more precise visualization of micro/nanobubbles in vivo. This technology enables clinicians to track bubble movement and drug delivery in real-time, improving the accuracy and efficacy of treatments [89]. Theranostic micro/nanobubbles, which combine therapeutic and diagnostic functions, are also an emerging area of research. These bubbles can deliver drugs and provide imaging contrast simultaneously, allowing for real-time monitoring of therapeutic efficacy and disease progression. Such dual-function bubbles have shown promise in preclinical studies, and clinical studies are underway [2]. Combining ultrasound-mediated drug delivery with other therapeutic modalities, such as immunotherapy and radiotherapy, is an area of growing interest. Preclinical studies have shown that combining these approaches can enhance therapeutic efficacy and overcome resistance mechanisms in tumors and neurodegenerative diseases [2]. The development of personalized medicine approaches using ultrasound-mediated drug delivery is another exciting area of research. By tailoring bubble formulations and ultrasound parameters to individual patient characteristics, clinicians can optimize treatment outcomes and minimize side effects [89].

## 7. Conclusions

Ultrasound-mediated drug delivery using micro/nanobubble technology holds great potential for revolutionizing the treatment of neurological diseases. Low-frequency ultrasound enables precise and safe delivery of therapeutic agents across the BBB, marking a key advancement in treating neurological disorders previously difficult to manage with conventional approaches. While essential clinical trials are underway, they represent just the initial phase of this groundbreaking technology. Advancing ultrasound-mediated drug delivery will require cross-disciplinary collaboration, optimized bubble technology, and strict adherence to FDA guidelines. Questions remain on the clinical utility across neurological disease and the long-term safety of repeated disruptions in the BBB. Despite existing challenges, the ability to safely and effectively target and deliver therapeutic agents across the BBB signifies a major advancement in treating these disorders, with ongoing research promising solutions for successful clinical implementation. By enhancing the precision and efficacy of drug delivery, this approach offers new hope for patients with conditions that are currently difficult to treat. With continued investment in research and clinical trials, the future of ultrasound-mediated drug delivery is bright, offering renewed hope for patients struggling with difficult-to-treat neurological conditions.

## Data Availability

Not applicable.
